# Extreme heat leads to short- and long-term food insecurity with serious consequences for health

**DOI:** 10.1093/eurpub/ckac080

**Published:** 2022-07-01

**Authors:** Carolin Kroeger, Aaron Reeves

**Affiliations:** Department of Social Policy and Intervention, University of Oxford, Oxford, UK; Department of Social Policy and Intervention, University of Oxford, Oxford, UK

*India’s move to ban wheat exports amid a record-breaking heat wave shows how local climate events may send shockwaves through global food security, but the debate around food supply and demand is missing a key ingredient—people’s capability to buy food.*

India is in the midst of an unprecedented heatwave with the highest May temperatures since records started 121 years ago.^1^^,^^2^ This extreme heat has the potential to drastically reduce access to food both in the next few weeks and over the coming years. Farmers and local officials estimate that the soaring temperatures will reduce yields by 10–50% this season and the food ministry slashed its output forecast by 6 million tons.^2^^,^^3^ The government has taken decisive short-term measures, banning all exports of wheat in a move to protect domestic supply and food security after global food prices increased by 40% since the start of the year and domestic food prices spiked.^1^ Analysts predict that the export ban will decrease global supply and increase prices even further, potentially spreading food insecurity across the globe.^4^

But the focus on wheat supply and demand in the debate around heat and food insecurity is missing a critical and more short-term dimension. India’s export ban may reduce food insecurity in the future, but it will not address people’s capacity to buy food tomorrow. Extreme heat not only damages agricultural yields and leads to supply drops and food insecurity in the long-term but also affects people’s short-term ability to generate income from labour and purchase food. In recent work in India, we show that this income-based mechanism mediates a link between short periods of heat exposure and higher short-term food insecurity with particularly acute effects among rural and low-income households.^5^

This crisis of short- and long-term food insecurity also has the potential to be a health crisis. Food insecurity increases the risk of depression and can in extreme cases lead to malnutrition, stunting, child mortality and higher adult mortality, including elevated risks of cardiovascular diseases.^6–9^

Doctors and hospitals have a key role to play. Medical professionals, many of whom will be a key part of India’s disaster response to heat waves, can advise their patients on the risks of heat-related food insecurity and refer patients to various forms of food support. Beyond the provision of immediate care, more research is also needed on how food insecurity might exacerbate the more commonly recognized health effects of extreme heat. This task requires both thinking through the interactions and downstream consequences of individual heat impacts, as well as disciplinary boundary crossing that considers climate science, public health and the political economy of hunger. Importantly, scientists, doctors and policymakers need to remain attuned to the uneven distribution of climate impacts and ensure that particularly vulnerable groups receive help.

India will not be the last country to experience the nexus of heat, health and food insecurity as climate change is forecast to increase the frequency and intensity of hot days across the globe.^10^ The path forward may not lie in protectionist trade reforms, but in varied policies that consider the different ways and interactions through which climate change affects food supply, demand as well as health and individual livelihoods.

## Funding

CK was funded by the Rhodes Trust. AR was funded in whole, or in part, by the Wellcome Trust 220206/Z/20/Z.

*Conflicts of interest*: None declared.
